# Editorial: Health literacy and digital health literacy among older adults: public health interventions

**DOI:** 10.3389/fpubh.2025.1764365

**Published:** 2026-01-14

**Authors:** Steven Hoffman

**Affiliations:** BYU School of Social Work, Brigham Young University, Provo, UT, United States

**Keywords:** digital health literacy, health literacy, intervention, older adults, public health

This Research Topic aims to examine health literacy and digital health literacy among older adults, with a specific focus on identifying challenges, disparities, and public health interventions that support healthy aging in an increasingly digital healthcare environment.

## Health literacy and digital health literacy among older adults: public health interventions

The world is experiencing an unprecedented demographic shift, with the global population of older adults projected to double by 2050. In parallel, digital technology has become a fundamental part of healthcare delivery, influencing health outcomes, social integration, and quality of life among seniors. However, the benefits of digital health tools are not equally accessible. Older adults face significant barriers in health literacy (HL) and digital health literacy (DHL), limiting their ability to access, evaluate, and utilize health information effectively. Public health interventions aimed at improving both HL and DHL among older adults are therefore essential for promoting healthy aging, managing chronic conditions, and reducing health inequities.

## Understanding health literacy and digital health literacy

Health literacy is broadly defined as the ability to obtain, understand, and use health information to make informed decisions. Digital health literacy extends this concept to digital environments, encompassing skills such as searching for, evaluating, and applying online health information, as well as navigating digital health platforms, apps, and telemedicine services (Mi et al.). Research shows that older adults often have lower levels of both HL and DHL compared to younger populations, due to factors including lower educational attainment, limited exposure to digital technologies, cognitive decline, and cultural or linguistic barriers (Fan et al.; Shao et al.).

For older adults with chronic diseases, low DHL can directly impact disease management. A study in China using latent profile analysis identified three distinct groups of older adults with chronic conditions based on their DHL levels: low, moderate, and high (Wang et al.). The low-literacy group had limited internet use, distrust of online health tools, and difficulty interacting with digital platforms, while the high-literacy group actively sought information, engaged with digital tools, and leveraged social support for health management. These findings highlight that older adults are not a monolithic group; interventions must be tailored to the specific capabilities and needs of each subgroup.

## Factors influencing digital health literacy

Digital health literacy among older adults is influenced by a combination of sociodemographic, technological, and social factors. Age, education, income, and urban vs. rural residence are strong predictors of DHL. Older adults with higher education and income tend to have greater confidence and skills in using digital health tools, whereas those in rural areas often face limited internet access and fewer opportunities for digital skill development (Cui et al.; Wang et al.).

Technological factors, such as access to multiple devices, familiarity with smartphones or computers, and attitudes toward the internet, also play a critical role. A scoping review found that older adults who regularly use digital devices and have positive attitudes toward technology demonstrate higher DHL, while those with limited experience or fear of making mistakes exhibit lower literacy (Wang et al.). Similarly, social support—including family encouragement, training programs, and community networks—can mediate the relationship between digital access and DHL, enhancing motivation and confidence (Cui et al.). Consistent with these findings, Kim et al. highlight that eHealth adoption among older adults is shaped by multilevel influences, including individual digital confidence, interpersonal support, and broader healthcare system factors, particularly during periods of rapid technological change.

Cultural and psychological factors are also relevant. For instance, the sense of empowerment gained from successful digital engagement can reinforce positive health behaviors, while concerns about privacy, fraud, or overreliance on technology may inhibit participation (Foo et al.; Wang et al.). Cognitive capacity, economic resources, and previous educational experiences further shape an individual's ability to engage with digital tools, underscoring the multifaceted nature of DHL determinants.

## Impacts of digital health literacy on health and wellbeing

High digital health literacy has demonstrable benefits for older adults, ranging from improved disease management to enhanced social participation. Studies show that seniors with greater DHL are more likely to engage in preventive health behaviors, utilize health services effectively, and maintain self-management routines for chronic conditions (Ma et al.; Shao et al.). For example, older adults who use online health information and telehealth platforms are better able to monitor chronic disease symptoms, adjust medications, and communicate with healthcare providers. The integration of DHL into self-management strategies is particularly crucial for older adults with comorbidities, who often require ongoing monitoring and support.

Beyond physical health, digital literacy influences psychological wellbeing and social integration. Internet use can reduce feelings of isolation, improve attitudes toward aging, and enhance cognitive functioning. Online engagement allows older adults to participate in virtual communities, maintain social networks, and access educational or recreational content, which collectively support mental health (Fan et al.; Wang). In the context of retirement migration, digital literacy promotes empowerment and social integration, while excessive use may pose risks such as smartphone addiction, highlighting the dual-edged nature of technology (Wang et al.).

Health behaviors are also shaped by peer effects, which are strengthened by internet use. Community health norms can influence individual behaviors, particularly in rural or collectivist settings, where older adults may rely on social networks for guidance (Cui et al.). By combining digital literacy with social engagement, interventions can amplify positive health behaviors and reduce disparities in self-care practices.

## Challenges and barriers to health literacy and digital engagement

Despite the benefits, older adults face numerous challenges in acquiring HL and DHL. A scoping review by Shi et al. further emphasizes that barriers to digital health literacy among older adults are multifactorial, including limited digital skills, low self-efficacy, accessibility issues, and insufficient age-appropriate training opportunities. The digital divide remains pronounced, particularly for individuals in middle-income countries, rural areas, or lower socioeconomic strata (Wang et al.). Limited access to devices, poor internet connectivity, and lack of formal training restrict engagement. Additionally, fear of scams, low confidence, cognitive decline, and health limitations impede effective use of digital health tools (Wang et al.; Foo et al.).

Measurement and research limitations further complicate interventions. Many existing DHL scales, such as eHEALS, focus on confidence rather than actual ability, and cross-sectional studies dominate the literature, limiting understanding of longitudinal effects (Wang et al.). There is also a lack of theoretically grounded, evidence-based interventions specifically designed for older adults. Without careful consideration of psychological, social, and technological factors, digital health programs risk reinforcing inequities or generating frustration among participants.

## Public health interventions for improving health literacy

Effective public health interventions must be multifaceted, combining education, training, technological access, and social support. Tailored programs should account for the diverse DHL profiles among older adults. For those with low literacy, interventions may focus on foundational digital skills, confidence building, and safe online practices. Moderate users can benefit from guided practice in using telehealth and mobile apps, while high-literacy seniors may require advanced modules to optimize their digital health engagement (Wang et al.).

Social support is a key enabler. Intergenerational programs, peer mentorship, and community-based workshops can provide hands-on guidance, foster motivation, and reduce isolation (Foo et al.; Gao et al.). Studies in Singapore demonstrate the effectiveness of pairing tech-savvy volunteers with seniors to facilitate learning and increase engagement in digital health tools. Similarly, Chinese retirement communities have successfully implemented training and support programs that promote digital inclusion while respecting cultural values, such as filial piety (Wang et al.). Supporting this approach, Wang et al. demonstrate that public perceptions of continuing care retirement communities in China are strongly influenced by trust, perceived quality of care, and access to supportive services, underscoring the importance of integrating digital and health literacy initiatives within community living environments.

Policy-level interventions are equally important. Ensuring equitable access to devices, internet connectivity, and health applications addresses systemic barriers to DHL. Governments and healthcare organizations can leverage digital platforms for health education, chronic disease management, and social engagement. Initiatives should also incorporate ethical and human-centered design principles to minimize risks of misinformation, ageist biases, and digital stress (Gao et al.; Foo et al.).

Integration of HL and DHL into healthcare delivery is another critical strategy. Health professionals can provide tailored guidance, encourage online information seeking, and monitor digital engagement to support self-management behaviors (Liu et al.; Shao et al.). Digital platforms, when combined with community programs, can foster both physical and mental wellbeing, enabling seniors to navigate the healthcare system more effectively.

## Implications for future research and practice

Evidence from large-scale epidemiological and informatics data during the COVID-19 period illustrates how access to timely, accurate digital health information is critical for older adults during public health crises, further reinforcing the need to strengthen digital health literacy capacities (Yu et al.). Future research should adopt longitudinal and mixed-methods designs to assess the long-term impact of DHL interventions. Updated, validated measurement tools are needed to capture both confidence and competence in digital health tasks. Interventions should be grounded in theoretical frameworks, such as social-ecological theory, self-determination theory, and active aging models, to address individual, social, and environmental factors comprehensively (Li et al.; Wang et al.).

Cultural considerations are essential. Interventions must respect local norms and values, such as attitudes toward family care and aging, while promoting inclusivity. Technology design should prioritize accessibility, usability, and ethical safeguards, ensuring seniors can safely engage with digital health platforms without exacerbating inequities or dependence.

Finally, leveraging the positive effects of internet use on social engagement, cognition, and health behaviors requires coordinated efforts across sectors. Community-based facilities, senior organizations, and healthcare providers can create environments that support both online and offline activities, enhancing the holistic wellbeing of older adults (Li et al.; Xiang et al.).

## Conclusion

The studies included in this Research Topic collectively demonstrate that health literacy and digital health literacy are critical determinants of health, self-management, and social integration among older adults. Across the included articles, older adults were shown to vary widely in their digital access, skills, and confidence, highlighting the need for tailored and context-specific public health interventions. The findings from this Research Topic emphasize the importance of combining education, social support, technological access, and policy-level strategies to reduce the digital divide. Together, these articles provide evidence-based insights that can inform future public health practice, research, and policy efforts aimed at empowering older adults to navigate health information and digital health tools more effectively.

